# Description of Ultrasound-Guided Lumbar Erector Spinae Plane (ESP) Block and Comparison of the Spread of Two Volumes of Dye in Cat Cadavers

**DOI:** 10.3390/ani15152157

**Published:** 2025-07-22

**Authors:** Adriana Vasconcelos Nobre, Heytor Jales Gurgel, Elaine Cristina Batista Torres, Geovana de Lima Aleixo, Daiara Joana Lima de Farias, Paulo de Souza Júnior, Roberto Thiesen

**Affiliations:** 1 Institute of Veterinary Medicine, Pará Federal University, Castanhal 68740-970, Brazil; adrivnobre@gmail.com (A.V.N.); jalesheytor@gmail.com (H.J.G.); torreselaine15@gmail.com (E.C.B.T.); geomedvet@gmail.com (G.d.L.A.); daiarajoana12@gmail.com (D.J.L.d.F.); 2 Uruguaiana Campus, Federal University of Pampa, Uruguaiana 97500-970, Brazil; paulosouza@unipampa.edu.br

**Keywords:** ESP block, regional anesthesia, felines, ultrasonography

## Abstract

The erector spinae plane (ESP) block is a widely used technique for perioperative analgesia. It involves the infiltration of anesthetics into the interfacial plane between the erector spinae muscle and the transverse processes of the vertebrae. In veterinary medicine, this technique has been adapted for different species, but there are no cadaveric studies in cats. This study describes the ultrasound-guided lumbar ESP-block technique and evaluates the spread of two volumes of dye in feline cadavers. Injections were performed at the second lumbar vertebra (L2) level using 0.6 mL/kg (high volume [HV]) and 0.4 mL/kg (low volume [LV]) of methylene blue bilaterally. After anatomical dissection, the distribution of the dye, the staining of the spinal nerve branches, and the presence of dye in the epidural space were recorded. The results demonstrated that the HV treatment provided wider longitudinal distribution, staining the dorsal branch of the spinal nerves in an average of five segments per injection, without extravasation into the epidural space. It is concluded that the HV treatment promotes efficient multisegmental spread in feline cadavers.

## Simple Summary

Local anesthesia techniques have been significantly advanced, largely due to the advent of ultrasound-guided blocks. One of these blocks is the erector spinae plane block, which is used for analgesia in spinal surgeries and in the treatment of acute and chronic pain that affects spinal column and epaxial muscles in both humans and animals. However, the lack of descriptive anatomical studies of this technique in cats limits the understanding of the anatomical differences between cats and other species, as well as potential associated complications. Thus, this study aimed to describe the erector spinae plane block in feline cadavers, investigating the distribution of two different volumes of dye to determine their spread. The larger volume presented a wider dye distribution, staining more spinal nerves without compromising important anatomical structures, such as the epidural space. These findings suggest that the erector spinae plane block, with the injection volume used, might be applied in spinal surgeries and for the treatment of acute or chronic pain. To confirm this hypothesis, additional studies are necessary to evaluate the technique in living cats.

## 1. Introduction

Locoregional blocks have gained considerable space in human and veterinary anesthesiology. In recent years, new techniques have been studied and implemented, and existing ones have been improved, partly due to technological advances in ultrasound equipment, which allow for the obtaining of better images, improving the visualization of the structures to be blocked. Among these new techniques, interfacial plane blocks stand out [1,2].

The erector spinae plane (ESP) block is a technique that was first described in human medicine in 2016 by Forero et al. [3] and, since then, has been adapted in veterinary medicine for different species. The block consists of infiltrating a local anesthetic into the interfacial plane formed by the erector spinae muscle complex and the transverse processes of the vertebrae, desensitizing the dorsal branches of the spinal nerves (DBSN) [4]. The spread pattern is not well determined. It might vary with the species and region of the column studied. In swine [5], the spread pattern is predominantly caudal to the injection site. In dogs, some studies report that there is no preferred pattern [1,6], while others observe a predominantly cranial distribution to the injection site [7]. This muscle complex extends from the cervical spine to the sacrum, which allows for the application of the blockade at any level of the spine, depending on the region to be desensitized [8].

The application of the ESP block may be beneficial in various clinical conditions, mainly in the control of acute pain resulting from trauma or surgery, as well as in the management of chronic neuropathic pain resulting from the DBSN [6,9,10]. In veterinary medicine, clinical trials suggest the importance of this blockade in perioperative analgesia in spinal surgeries in dogs. Additionally, the blockade may contribute to the reduction of analgesics and general anesthetics requirements, therefore decreasing cardiovascular complications related to these drugs [11,12,13,14,15,16].

Cadaveric studies that describe the ESP block are essential to detect possible complications for later application in clinical studies [1,17]. There are studies describing this technique on cadavers of humans, dogs, horses, pigs, and cows [5,6,18,19,20]. However, cadaveric studies in cats were not found in the literature reviewed. There is, however, a case series recording the application of the technique in a lumbar approach in three cats that underwent spinal surgeries. The study reports that all animals presented hypotension, and one cat presented apnea shortly after the blockade was performed [21].

Therefore, although the ESP block is a blockade with potential benefits, it should be considered that there is still a lack of studies, and that it is a recent technique with complications that still need to be elucidated to improve the accuracy and safety in the execution of the blockade [8,22]. In addition, there are significant anatomical differences between species in veterinary medicine, so it is essential to perform studies about this blockade in different species.

Thus, the present study aims to describe the anatomy of the lumbar ESP and evaluate the spread of two volumes of dye and the number of DBSN stained in feline cadavers.

## 2. Material and Methods

### 2.1. Animals

The study was performed on eight cadavers of cats donated by hospitals and clinics in Castanhal, Pará, Brazil (Supplementary file: cadaver donation term). The sample size was defined based on similar cadaveric studies in other species [5,6]. The cat died from different causes that were not related to the proposed study. Only cadavers of animals that did not present any disease along the entire dorsal musculature and spinal column, based on their medical records and physical examination, were included in the study. Imaging exams were not performed for this purpose. Only cadavers that had died no more than 2 days before and were kept frozen were accepted for the study to minimize interference of the cadaver’s integrity in the execution of the blockade and dissections. There was no selection of animals according to sex, age, body score, or breed. However, all corpses included were animals 2 to 4 years old. Approval from the ethics committee of our institution is not required for cadaveric investigations involving animals euthanized for unrelated reasons.

### 2.2. Ultrasound-Guided Injections

**Figure 1:**
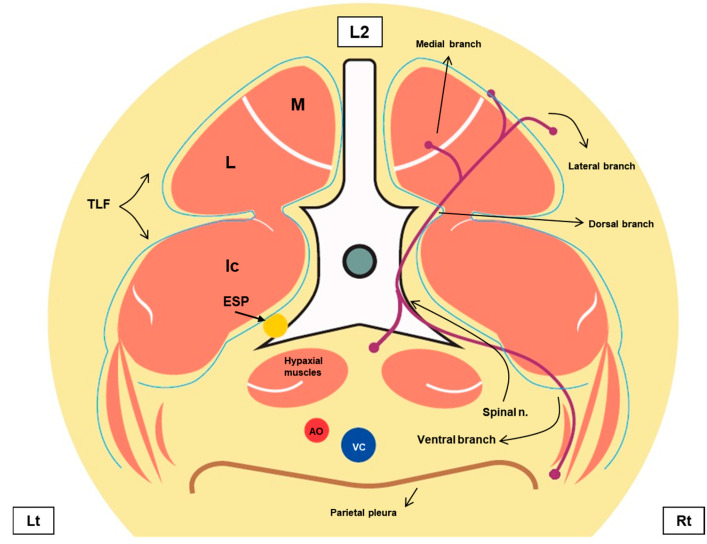
Schematic representation of the lumbar spine region at the level of the second lumbar vertebra (L2). The different layers of the thoracolumbar fascia (TLF) are represented in light blue. The spinal nerve and its dorsal, ventral, lateral, and medial branches are represented in purple. The yellow circle shows the final injection point of the erector spinae plane (ESP) block. Ic: m. iliocostalis lumborum; L: m. longissimus lumborum; M: m. multifidi; AO: aorta; VC: vena cava; Lt: left; Rt: right. Source: personal archive.

The animals included in the research remained frozen and, on the day before the study, were thawed for subsequent ultrasound-guided injections. The injections were performed at the level of the second lumbar vertebra (L2) in each cat using Methylene Blue (1%, Perfyl Tech Reagente e Soluções, São Bernardo do Campo, SP, Brazil) in two volumes: 0.6 mL/kg for high-volume treatment (HV) and 0.4 mL/kg for low-volume treatment (LV). Each cat received both volumes, with HV treatment on the right side and LV on the left side of the spine. Anatomical structures relevant to the study are shown in Figure 1.

For the injections, the cadavers were positioned in sternal recumbency, with the forelimbs extended cranially and hindlimbs extended caudally. The hair was clipped over the entire dorsum, from the thoracic to the sacral spine, along with the lateral abdomen bilaterally. For the correct detection of the puncture point, the vertebra L2 was identified by palpation of the spinal processes, counting from the last lumbar vertebra (L7) and successively marked with a permanent marker.

The ultrasound device used was the Logiq V2 (GE Healthcare, Chicago, IL, USA), with a linear transducer (L6-12 RS, 6 to 12 MHz; GE Healthcare, USA). The ESP injection approach was adapted from that described by Medina–Serra et al. [17]. The transducer was positioned at the level of L2, parallel to the dorsal midline in a parasagittal plane until the identification of the lateral border of the transverse process (TP) of L2, identified as a hyperechoic convex line with posterior acoustic shadowing. Also, muscular planes and TPs of the first and third lumbar vertebra (L1 and L3, respectively) were identified. The transducer was slowly moved until a clear image of the interfacial plane and TPs was obtained.

A 21-gauge, 50 mm insulated needle (PHBR, São Paulo, SP, Brazil) was used. The needle was inserted in-plane in a caudoventral direction, with a clear image of the target point. The needle was slowly advanced until it reached the dorsal surface of the target TP. The correct positioning of the needle in the interfacial plane was confirmed by the ultrasonographic visualization of the tip of the needle in contact with the dorsal aspect of the TP of L2. All injections were performed by the same investigator, who was right-handed. The depth of the target plane from the skin surface, the injection time and its longitudinal propagation, and the quality of the image generated in ultrasound were recorded.

### 2.3. Anatomical Study and Dye Distribution Evaluation

Dissection was performed immediately after injection. The dissection protocol was based on and adapted from that described by Röhrmann et al. [23] and Medina–Serra et al. [17]. Cats were initially positioned in sternal recumbency. Briefly, the skin was incised along the dorsal midline above the spinous processes of the lumbar vertebrae in a craniocaudal direction. The layers of skin, subcutaneous fat, and thoracolumbar fascia were reflected laterally to expose the musculature of the erector spinae complex (m. multifidi, m. iliocostalis lumborum, and m. longissimus lumborum). The m. iliocostalis lumborum and m. longissimus lumborum muscles were displaced ventrolaterally to visualize the m. multifidi and expose the DBSN as they emerge from the lumbar intervertebral foramen to branch off the ESP complex. This dissection was performed in the caudal and cranial directions to assess the dispersion and quantification of stained DBSN, and the vertebrae reached. The DBSN was considered stained when the dye solution was identified around the branches.

The cadavers were then placed in dorsal recumbency, and a ventral plane dissection was performed to assess eventual staining and dye distribution in the hypaxial muscles and ventral branches. An incision was made in the ventral midline to access the thoracic and abdominal cavities, with subsequent *en bloc* removal of all organs for better visualization of the dorsal abdominal wall. It was assessed whether the sublumbar, intercostal, abdominal muscles, and ventral branch were stained. Finally, a cross-section was made in all stained vertebrae with a bone saw to assess eventual migration of the dye to the epidural space and the staining in the epaxial and hypaxial muscles.

### 2.4. Statistical Analysis

The Shapiro–Wilk test was performed to assess the normality of the values obtained. Subsequently, a *t*-test was used to compare the number of DBSN stained with LV and HV. Differences were considered significant at a value of *p* < 0.05. Data were analyzed using GraphPad Prism Version 10.2.2 (GraphPad Software Inc., Boston, MA, USA).

## 3. Results

### 3.1. Anatomical Findings

The present study was performed in eight adult cats, with an average weight of 3.9 ± 1 kg, totaling 16 injections.

In the anatomical study, after macroscopic dissections and cross-sections, it was observed that the thoracolumbar fascia forms a dense layer with fibroelastic characteristics that surrounds the muscles of the erector spinae complex and the TPs. Among these muscles, the m. longissimus lumborum and the m. iliocostalis lumborum merge into a single thick muscle, being innervated by the DBSN. It was noted that the DBSN exits the intervertebral foramen and inserts diffusely into the muscle group, making it hard to evaluate macroscopically. In our study, it was not possible to differentiate the lateral and medial branches of the dorsal nerves.

The thoracolumbar fascia also separates the epaxial muscles from the hypaxial muscles, as was observed in the dissections in the ventral plane. Similar to the DBSN, the ventral branches of the spinal nerves insert into the sublumbar muscles.

### 3.2. Sonoanatomy of ESP Block Injections

**Figure 2:**
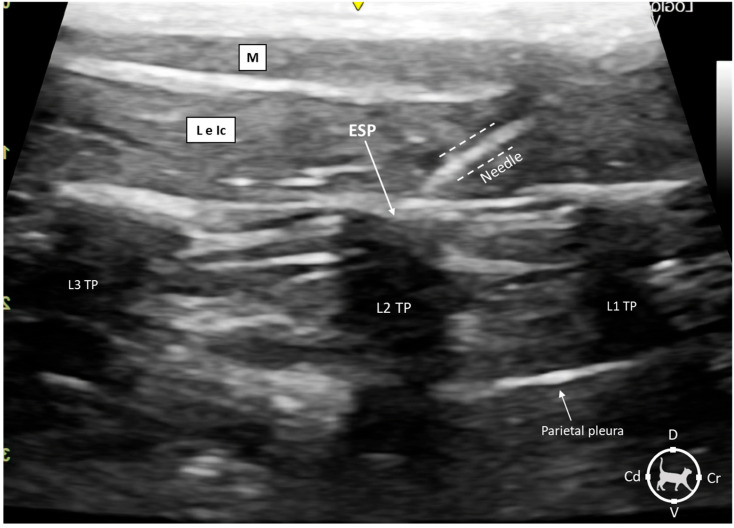
Sonoanatomy of the epaxial muscles and landmarks for injecting the erector spinae plane (ESP) in the lumbar region in a feline cadaver. The injection target was the ESP at the level of the transverse process of L2, with the target point centered in the image. The cat is positioned in sternal recumbency with the ultrasound transducer longitudinally in a parasagittal orientation. The dotted lines identify the needle beam. ESP: erector spine plane; L3 TP: transverse process of the third lumbar vertebra; L1 TP: transverse process of the first lumbar vertebra; L and Ic: m. longissimus lumborum and m. iliocostalis lumborum; M: m. multifidi; Cd: caudal; Cr: cranial; D: dorsal and V: ventral.

It was possible to visualize the sonoanatomy of the spine and its structures relevant to the execution of the technique in all animals, identifying the TP of L2. The thoracolumbar fascia was observed as a hyperechoic line, clearly demarcating the erector spinae muscle complex. The tip of the needle is also visualized (Figure 2).

All punctures were performed in a cranial position to L2, advancing the needle in a caudoventral direction until the point of injection. During the injection, it was possible to visualize in real time the craniocaudal hydrodissection effect caused by the injection. The images clearly showed the formation of an anechoic horizontal pocket that disclosed the interfascial plane of the erector spinae. Craniocaudal hydrodissection was longer in the HV treatment compared to the LV treatment. There was no perforation of the parietal pleura in any of the injections performed.

The needle was introduced 3.4 ± 0.24 cm from the skin, although this depends on the insertion point and the angle of the needle to the skin. Structure visualization was possible with a frequency of 8 MHz, except for two animals, where an adjustment to 12 MHz was necessary.

### 3.3. Dye Spread

**Figure 3:**
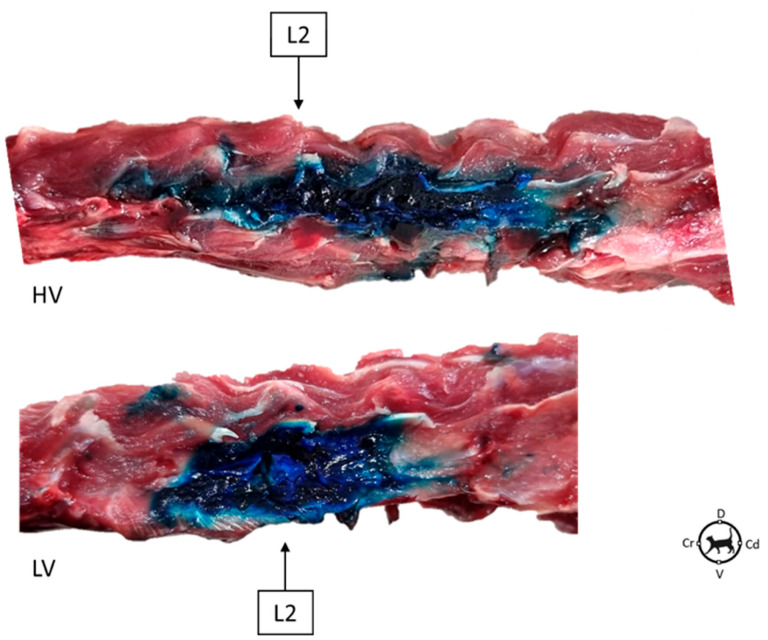
Anatomical dissection of the spine comparing dye distribution and range of stained vertebrae after ultrasound-guided injection into the erector spinae plane (ESP) at L2 level in the two treatment volumes: high volume (HV) and low volume (LV). In blue, lateral aspect of the vertebral bodies. Cd: caudal; Cr: cranial; D: dorsal and V: ventral.

**Figure 4:**
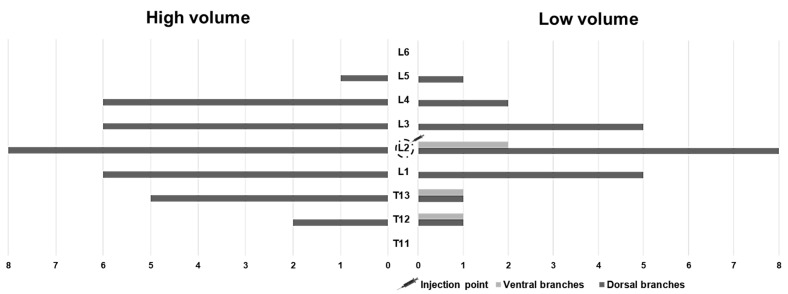
Number and level of dorsal and ventral branch stained with dye solution after erector spinae plane (ESP) injections at L2 using two injection volumes in eight cat cadavers. High volume: 0.6 mL/kg; low volume: 0.4 mL/kg. L, lumbar vertebra; T, thoracic vertebra.

The DBSN pathway was difficult to assess due to the disruption of adjacent tissues during dissections to expose the nerves. In all animals, epaxial muscles and DBSN were stained to a greater extent in the HV treatment when compared to the LV (Figure 3). The number of vertebral bodies stained that correspond to the number of DBSNs stained in the HV treatment was 4.5 ± 1.2, while in the LV treatment, it was 2.8 ± 1.3. The HV treatment stained significantly more DBSN than the LV treatment (*p* = 0.01; Figure 4).

In both treatments, dispersions occurred outside the target plane. In the LV treatment, three injections showed distribution ventromedial to the TP, resulting in staining of the hypaxial muscles and ventral branch, with two injections distributing at the level of L2. The third injection in LV showed a different dispersion pattern, staining the ventral branch and hypaxial muscles at the level of the T12 and T13 vertebrae, in addition to staining the 13th internal intercostal muscle. In the HV treatment, two injections stained the hypaxial muscles without reaching the ventral nerves. In one injection of the HV treatment, methylene blue was observed within the mm. iliocostalis and hypaxial, lateral to L2.

**Figure 5:**
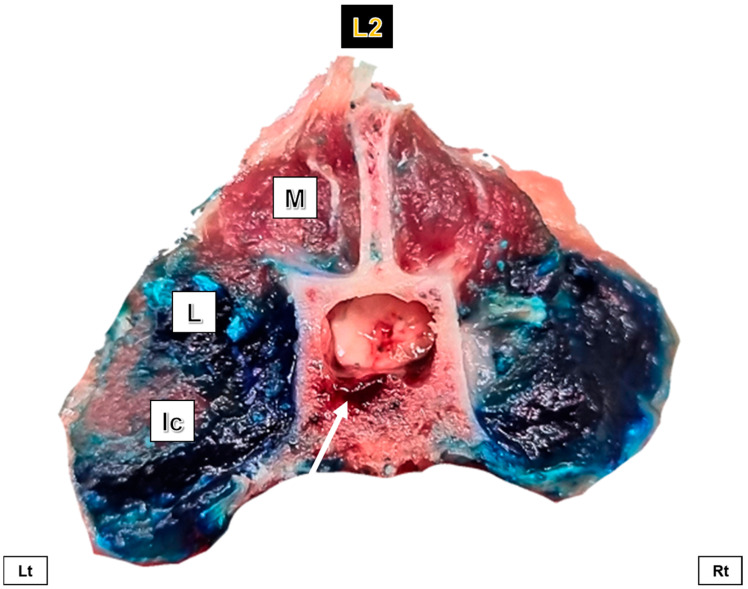
Cross-section of the second lumbar vertebra (L2) showing the distribution after an ultrasound-guided injection into the erector spinae plane (ESP) in both volumes; LV on the left (Lt) and HV on the right (Rt). In blue, the dye staining the m. iliocostalis lumborum (Ic) and m. longissimus lumborum (L) muscles. There was no dye migration into the epidural space (white arrow). M: m. multifidi.

There was no migration to the epidural space in any injection. The dye followed a linear distribution parallel to the spine within the ESP muscles, staining mainly the m. longissimus lumborum and the m. iliocostalis lumborum in its entirety (Figure 5).

## 4. Discussion

This study proposes the first description of the ultrasound-guided lumbar ESP block technique in feline cadavers through their sonoanatomy and anatomical description, with an injection point at the L2 level. The study demonstrates the feasibility of performing an ultrasound-guided injection in the interfascial plane of the lumbar ESP using the longitudinal approach in feline cadavers. The results of this study demonstrate that the HV treatment (0.6 mL/kg) results in a better longitudinal distribution of the dye, compared to LV, staining the DBSN in an average of five spinal segments.

The injection volumes selected for the different treatments were determined based on clinical applicability, taking into account the toxic doses of local anesthetics, similar to what has been performed in other studies of ultrasound-guided blocks in animals [1,5,17]. However, 0.6 mL/kg of bupivacaine 0.5% would be equivalent to 3 mg/kg of bupivacaine. To date, there is only one paper recording pharmacokinetics of bupivacaine for fascial plane injection in cats [24], which states that a safe dose comprises 2.5 mg/kg in healthy cats. So, diluting the drug could be an option to avoid surpassing the dose and any toxic effect.

Statistical analysis revealed a significant difference between the treatments in this study, differing from the results of Portela et al. [1], who described ultrasound-guided ESP blocks in the thoracic spine of dogs. These authors hypothesize that their results occurred due to a low sample and a large volume of dye injected into the m. longissimus thoracis, which may have led to wasted volume and poor distribution. In contrast, our results corroborate Ferreira et al. [6], who also applied the technique to the thoracic spine of dogs and obtained a greater number of stained vertebral segments (eight) with a higher volume (1 mL/kg). It must be considered, however, that both experiments used dog cadavers, and anatomic differences may have played a role in the spread pattern.

Three injections of the LV group resulted in the dye spreading in the ventromedial and ventrolateral aspects of the vertebra, reaching the hypaxial muscles, ventral branch of the spinal nerve, and intercostal muscles. After the dissections and cross-sections, the authors did not identify a visible passage between the epaxial and hypaxial compartments that would explain this extravasation. Some hypotheses may be considered: two of the cadavers were in a poor conservation condition, which may have favored inadequate migration to the ventral structures and the possibility of failure during the execution of the technique, where the methylene blue may have been injected ventral to the thoracolumbar fascia.

Traditionally, the ESP block is used to desensitize the DBSN. However, studies in humans suggest that the ESP block can desensitize structures innervated by the ventral branch of the spinal nerves, making it an alternative for blocks in thoracic and abdominal surgeries [3,8,18]. The mechanism for the blockade of the ventral branch is still controversial. Some reports suggest that there is communication between the epaxial and hypaxial compartments due to incomplete closure of the intertransverse space, allowing the spread of the solution injected. However, the exact pathway has not yet been determined [8,25,26].

The present study performed the traditional longitudinal approach of the ESP block technique. It is valuable to consider the fact that the ESP block can be performed at any level of the spine. This demonstrates that its mechanism of action is not fully comprehended due to the variable anatomical patterns along the spine [27]. With this in mind, new approaches have been described to improve the blockade according to the target region of the spine. The traditional approach consists of positioning the transducer in a longitudinal or parasagittal plane, with the anatomic landmark for injection being the dorsal edge of the lateral end of the transverse process [18]. However, recent studies in humans and dogs suggest a new approach in the transverse plane, where the mammillary and accessory processes are used as anatomic landmarks for positioning the needle and applying the local anesthetic. Some authors suggest that the traditional approach is more appropriate for the thoracic spine, while the transverse approach is more appropriate for the lumbar region [7,9,17,28]. However, comparative studies between these techniques in cats are necessary to define the best approach considering the anatomical particularities of each vertebral segment in this species.

Similarly to dogs, the lumbar TPs of cats are oriented in a cranioventral direction. Cats also have seven lumbar vertebrae with PTs and vertebral bodies longer than the thoracic vertebrae [17,23]. However, they are visibly smaller compared to dogs [23]. These particularities of the feline lumbar segment may affect the distribution of the injectate within the ESP compartment and around the spinal branches, reinforcing the necessity of further studies of this technique in cats.

Most cases of ESP block application in clinical and surgical routines are in multimodal perioperative analgesia protocols for spinal surgeries since the goal is to desensitize the DBSNs [10,26,29]. In the present study, in all injections performed, in both treatments, there was dye spreading along the DBSNs, but not along structures such as the epidural space, pleura, and blood vessels as aorta and vena cava, suggesting that there was no vascular distribution. Therefore, this fact suggests that this block could provide appropriate analgesia for procedures at the lumbar spine level, such as hemilaminectomies, in cats.

In addition, the ESP block has been an alternative to traditional blocks, such as epidural and paravertebral blocks, classic techniques frequently used in thoracic, spinal, and abdominal surgeries [1,8]. The choice of the ESP block in these cases may be motivated by the fact that it is a technique simple to perform and the consideration of its safety since it is administered at a safer distance from important structures, such as the spinal cord, pleura, and vascular structures [2,26,29,30].

This study chose to determine the side of the application of each treatment volume to standardize the injections in the cadavers and avoid mistakes during the analysis of each injected volume. However, this may be considered a limitation of our study, as for a right-handed operator, it is easy to perform the block on the left side of the spine. Trying to mitigate this, for both injections in the present study, performed by a right-handed operator, the cadavers remained at the same position, with the operator performing the block at the right side of the spine shifting and positioning the ultrasound probe and the needle over the midline of the spine. The fact that the investigators were not blind to the treatment may also be considered a limitation.

Another limitation is regarding the characteristics of tissues in cadavers, which differ significantly from those in live animals due to altered tissue integrity and physiological processes [1] as they are hard to predict the effect of cadaveric tissue over the dye spreading [8]. Thus, injections in cadavers are only an approximation of what occurs in live individuals. Regarding the frozen–thawing cycle, there is an impact on tissue integrity according with the number of cycles performed, which leads to an increase in disrupted cells, increasing the size of the extracellular area arising from the releasing of intracellular components [31]. So, ideally, for educational purposes, the frozen–thawing cycle should be performed only once. Therefore, the use of fresh frozen cadavers thawed only once, such as those used in the present study, may present less alterations in tissue integrity. Even so, dye spreading is not equivalent to what happens in live animals [8].

The lack of imaging studies to certify the absence of spine and muscular pathologies before the injections and for the analysis of the distribution of the injectables, either by radiography or computed tomography, is another limitation. The reason for this obstacle was the lack of available equipment for the research.

## 5. Conclusions

This study describes the ultrasound-guided ESP block technique in feline cadavers, where high-volume treatment (0.6 mL/kg) showed better multisegmental propagation than low-volume treatment (0.4 mL/kg). Ultrasound-guided ESP injections are technically simple to perform and result in a consistent distribution of the injectates along the interfascial plane of the ESP, which runs the dorsal branch of the spinal nerves. Thus, the study demonstrates that this technique is feasible in the longitudinal approach at the lumbar level in feline cadavers, achieving a better craniocaudal longitudinal distribution by staining the dorsal branches of spinal nerves in an average of five spinal segments.

## Data Availability

Data are contained within the article and Supplementary Materials.

## Supplementary Materials

The following supporting information can be downloaded at: https://www.mdpi.com/article/10.3390/ani15152157/s1, Cadaver Donation Term (TERMO DE DOAÇÃO DE CADÁVER E OU PEÇAS ANATÔMICAS)
