# Supplementary material for: Description of Ultrasound-Guided Lumbar Erector Spinae Plane (ESP) Block and Comparison of the Spread of Two Volumes of Dye in Cat Cadavers
Source: Animals (Basel). 2025 Jul 22;15(15):2157. doi: 10.3390/ani15152157 (PMC12345535; doi:10.3390/ani15152157)
Supplement: Supplementary file 1 [file animals-15-02157-s001.zip › animals-3266608-supplementary.pdf]

TERMO DE DOAÇÃO DE CADÁVER E OU PEÇAS ANATÔMICAS

DADOS DE ORIGEM DO MATERIAL	
Pessoa Jurídica ( ) Pessoa Física ( )	
Nome do Estabelecimento/Instituição:	
Nome do Responsável:	
CNPJ ou CPF:	
Endereço:	
Bairro:	Cidade:
Estado:	CEP:
Telefone 1: ( )	Telefone 2: ( )
E-mail:	
**DESCRIÇÃO DO MATERIAL**	
Assinale o tipo de material:<br>( ) Cadáver do Animal ( ) Peça anatômica ou fragmento de tecidos	
Quantidade:	
Método de eutanásia:	
Causa morte:	
Espécie:	
Raça:	
Idade:	
Características externas que identificam o animal:	
Método de identificação:	

Eu, ________________________________ acima qualificado, declaro que estou doando o material descrito ____ a Universidade Federal do Pará - UFPA, o qual será destinado a _______________________________________ do curso de _____________________________ em __________________________________, a fim de que possam ser utilizados no auxílio da formação profissional do corpo discente da UNOESC.

Castanhal, _____ de _____________ de _______

____

Responsável

Assinatura e carimbo (se houver)
